# Temporal Patterns of Breast Cancer-Related Sickness Absence and Social Insurance Expenditures Among Women in Poland, 2020–2024: A Nationwide Registry-Based Study

**DOI:** 10.3390/curroncol33060326

**Published:** 2026-06-01

**Authors:** Piotr Artur Winciunas, Justyna Grudziąż-Sękowska, Agnieszka Mazurek, Kuba Sękowski, Wojciech S. Zgliczyński, Mateusz Jankowski

**Affiliations:** School of Public Health, Centre of Postgraduate Medical Education, 01-826 Warsaw, Poland

**Keywords:** breast cancer, sickness absence, absenteeism, social insurance, women’s health, productivity loss, indirect costs, work disability, nationwide study, Poland

## Abstract

**Background/Objectives**: Sickness absence due to breast cancer generates significant indirect costs. This nationwide registry-based study aimed to evaluate temporal patterns and the population-level burden of sickness absence, rehabilitation benefit utilization, and social insurance expenditures related to breast cancer among women in Poland between 2020 and 2024. **Methods**: This nationwide analysis is based on the Social Insurance Institution registry. Data on females unable to work due to breast cancer were analyzed (ICD-10: C50). Short-term sickness absence was analyzed. **Results**: The number of sick leave days due to breast cancer varied from 1,241,223 in 2021 to 1,444,863 in 2024. The number of sick leave certificates increased every year: from 55,732 in 2020 to 79,979 in 2024. The most dynamic growth between 2020 and 2024 occurred in the 45–49 group, where sick leave days increased +33.4% and certificates increased +70.6%. There were significant regional differences. The sickness absence days per certificate due to breast cancer decreased over time, from 23.5 days in 2020 to 18.1 days in 2024. The total number of medical certificates on rehabilitation benefit due to breast cancer varied from 4811 in 2021 to 5281 in 2024. Social insurance expenditures on sickness absence varied from 112,064.9 thousand PLN (approximately 25.24 million EUR) in 2020 to 207,687.9 thousand PLN (approximately 48.3 million EUR) in 2024 and expenditures on rehabilitation benefits varied from 56,812.8 thousand PLN (12.8 million EUR) in 2020 to 89,692.6 thousand PLN (20.83 million EUR) in 2024. **Conclusions**: This population-based registry study revealed a high economic burden of sickness absence due to breast cancer in Poland.

## Simple Summary

Breast cancer-related sickness absence generates substantial indirect costs for social insurance systems. This nationwide study assessed trends in sickness absence among women in Poland using Social Insurance Institution registry data for 2020–2024. The number of sick leave certificates increased steadily from 55,732 in 2020 to 79,979 in 2024, while total sick leave days ranged from 1.24 million in 2021 to 1.44 million in 2024. The most dynamic growth occurred among women aged 45–49 years. Despite the rising number of certificates, the sickness absence days per certificate decreased over time. Social insurance expenditures almost doubled during the study period. These findings may inform return-to-work planning, occupational rehabilitation, and coordinated care pathways linking oncology services, occupational medicine, primary care, and the social insurance system to support women during and after treatment.

## 1. Introduction

Breast cancer is the most commonly diagnosed cancer in women [1,2,3]. This type of cancer is also a leading cause of cancer-related morbidity and mortality among women globally [1,2,3]. In 2022, breast cancer was diagnosed in 2.3 million women worldwide (24% of all diagnosed neoplasms) [2,4]. Moreover, 670 thousand deaths attributed to breast cancer were reported in 2022 (15% of all cancer-related deaths) [2,4].

Poland is a European Union country with a high burden of breast cancer incidence and mortality [5,6,7]. According to the National Cancer Registry [7], between 2013 and 2022, an average of 19,250 new cases of breast cancer in women were recorded annually in Poland. In 2022, 21.5 thousand newly diagnosed cases of breast cancer and 6.6 thousand deaths related to breast cancer were reported in Poland [7]. In Poland, breast cancer is responsible for one-quarter of all cancer cases in women and 1/8 of cancer-related deaths [7]. In 2022, most of the newly detected breast cancer cases were among women aged 50–69 years (48.7%), 29.9% of newly detected cases were in women aged 70 years and over, 19.6% were in women aged 34–49 years, and 1.8% were in women under 35 years [7]. Breast cancer is typically diagnosed in older women—the median age of onset in women is 63 [3,7]. The earliest cases are usually diagnosed around age 25, but 0.1% of breast cancer cases also occur in even younger women [7].

Breast cancer is the subject of population screening aimed at early detection of the disease [8,9]. As part of the population screening program financed from public funds in Poland, women aged 45–74 can have a mammogram every two years [8,9,10]. On 1 November 2023, the age criterion changed, and the group eligible for mammography screening under the program was expanded from 50 to 69 years to 45–74 years [8,9]. As of 1 February 2026, the percentage of the eligible population who had used the mammography-based breast cancer screening program in Poland was 32.29% [10].

The majority of newly diagnosed breast cancer cases in Poland are early-stage breast cancer, i.e., cancer limited to the mammary gland or lymph nodes [7,11]. Early detection and timely diagnosis improve prognosis and expand therapeutic options [11].

The management of breast cancer has evolved substantially and currently includes multimodal approaches combining surgery, radiotherapy, and systemic therapies such as chemotherapy, endocrine therapy, targeted therapies (e.g., anti-HER2 agents, CDK4/6 inhibitors, PARP inhibitors), and immunotherapy. These treatments differ in duration, toxicity profiles, and impact on patients’ functional status and ability to work [12,13].

High incidence of breast cancer leads to high direct medical costs of treatment but also generates substantial indirect costs due to temporary work incapacity [14,15]. In Poland, sickness absence is compensated within the social insurance system. Under the social insurance, working individuals can receive social benefits in the form of short-term or long-term sickness benefits, depending on the duration of incapacity for work. Sickness absence due to breast cancer may depend on various factors, including stage at diagnosis, treatment type (surgery, chemotherapy, radiotherapy), access to timely care, healthcare system organization, rehabilitation, and workplace-related factors [11,12].

From 1 December 2018, doctors in Poland have issued sick leave certificates only in electronic form, which are recorded in a dedicated IT system managed by the Social Insurance Institution [16]. The social insurance registry in Poland provides standardized, nationwide data, among other things, about sickness absence causes, duration (absence days), number of certificates issued, as well as the gender and age of the person to whom the certificate was issued [16]. Population-based data on sickness absence due to breast cancer may inform clinicians and policymakers on about impact of new treatment methods and organizational standards on breast cancer care, as well as support health system planning. However, there is a lack of population-based studies on sickness absence in Poland.

Therefore, this nationwide registry-based study aimed to evaluate temporal patterns and the population-level burden of sickness absence, rehabilitation benefit utilization, and social insurance expenditures related to breast cancer among women in Poland between 2020 and 2024.

## 2. Materials and Methods

### 2.1. Data Source and Measures

This was a descriptive observational study based on aggregated nationwide administrative registry data obtained from the Social Insurance Institution in Poland.

The data was obtained from the Department of Statistics and Actuarial Forecasts of the Social Insurance Institution (pol. Zakład Ubezpieczeń Społecznych) [16], as part of a public information request—public statistics for scientific analyses. In accordance with the Polish social insurance system, short-term benefits (sick leave benefits and rehabilitation benefits) from the sickness fund were analyzed. Within the social insurance system, certificates entitling the holder to benefits are issued by doctors as part of medical certification.

A sick leave benefit is a social insurance payment granted to employees who are temporarily unable to work due to illness or injury [16]. It is intended to partially compensate for lost earnings during periods of medically certified incapacity for work. Temporary incapacity for work is certified by a medical doctor via a ZUS ZLA certificate (sick leave medical certificate) [16]. The sickness benefit usually covers partial salary replacement (typically 80% of the base salary). A full base salary is paid in specific cases such as hospitalization or an occupational accident. After this period, the benefit may continue under the rehabilitation benefit or a disability pension.

The rehabilitation benefit is a short-term social insurance payment provided to insured individuals who are still unable to work after exhausting the standard sick leave benefit (ZUS ZLA). This benefit aims to prevent permanent disability as well as facilitate a return to work; it is offered to support the insured during a prolonged period of incapacity and facilitate medical or occupational rehabilitation. The rehabilitation benefit is granted for a maximum of 12 months following the end of the standard sick leave [16]. The payment is usually 80% of the previous sickness benefit (adjusted according to regulations).

A medical certificate for sick leave is issued by a physician (general practitioner or specialist) authorized to certify incapacity for work in Poland. A medical certificate for the rehabilitation benefit is issued by a dedicated medical examiner of a social insurance institution—a medical doctor, who is an employee of the Social Insurance Institution [16]. In Poland’s medical certification system, the cause of incapacity for work is determined by a physician based on the International Classification of Diseases (ICD).

In this study, data on all females unable to work due to breast cancer were analyzed. The analysis included all women receiving a sick leave benefit or a rehabilitation benefit due to ICD-10 code C50. Analyses regarding sick leave included the number of sick leave certificates issued in a given year, the number of days of sickness absence in a given year, the sickness absence days per certificate, and social insurance expenditures (in PLN thousand) on sickness absence. Data on the rehabilitation benefit included the number of medical certificates due to breast cancer (ICD-10 code C50) issued at the national and regional levels. In the case of conversions from PLN to EURO, the average annual exchange rate according to the National Bank of Poland was used (2020: 1 EUR = 4.44 PLN; 2024: 1 EUR = 4.30 PLN) [17].

Each sick leave certificate was treated as a separate administrative event. The dataset did not allow the reconstruction of continuous episodes of sickness absence at the individual level; therefore, certificate counts should not be interpreted as counts of distinct patients or independent clinical episodes.

The total duration of sickness absence represents the sum of all certified days within a given year, regardless of whether certificates were issued as initial or continuation forms.

Data were analyzed only for women, because sick leave in men diagnosed with C50 constitutes approximately 0.5% of all sick leave for this reason, so these groups were excluded from the analysis.

The study protocol was reviewed and approved by the Ethics Committee at the Center for Medical Postgraduate Education, Warsaw, Poland: approval number 89/2025 as of 8 October 2025.

### 2.2. Statistical Analysis

Descriptive statistics were used. Data were analyzed using IBM SPSS v. 29. Data were analyzed by age group and administrative region (voivodeships). Rates per 100,000 women were calculated using the total female population in each age group and region according to Statistics Poland, rather than the insured or employed population. Therefore, these values represent population-level indicators and are not direct measures of individual risk among insured workers. Population denominators used for rates per 100,000 women were based on the total female population in each age group and region according to Statistics Poland data, not exclusively the insured or employed women [18]. Therefore, the reported rates should be interpreted as population-level indicators rather than direct measures of risk among insured workers. Demographic data on the population size in each age group and region were obtained from the demographic yearbooks published by the Central Statistical Office [18]. The absolute number of medical certificates granting entitlement to the rehabilitation benefit due to breast cancer was presented.

Due to the aggregated nature of the registry data, measures of central tendency, such as the median/IQR could not be calculated at the individual level.

At the regional level, data were presented for women aged 40–59 years, as the risk of breast cancer significantly increases after 40 years of age and the retirement age for women in Poland is 60 years [11,16].

Because the study was based on aggregated nationwide administrative data representing the entire population of insured women registered in the Social Insurance Institution database rather than a sampled population, confidence intervals were not calculated for the descriptive indicators presented. Due to the descriptive and aggregated nature of the registry data, the analyses were intended to characterize temporal patterns rather than establish causal relationships.

## 3. Results

The number of sick leave days due to breast cancer varied from 1,241,223 in 2021 to 1,444,863 in 2024 (Table 1). The number of sick leave certificates increased every year: from 55,732 in 2020 to 79,979 in 2024. Overall, between the years 2020 and 2024, there was a modest increase in the absolute number of sick leave days (+10.2%), alongside a much greater increase in the absolute number of sick leave certificates (+43.5%) (Table 1). The largest absolute numbers of both sick leave days and certificates across all years were observed among women aged 45–49, 50–54 and 55–59. The most dynamic growth between 2020 and 2024 occurred in the 45–49 group, where sick leave days increased +33.4% and certificates increased +70.6% (Table 1).

**Figure 1:**
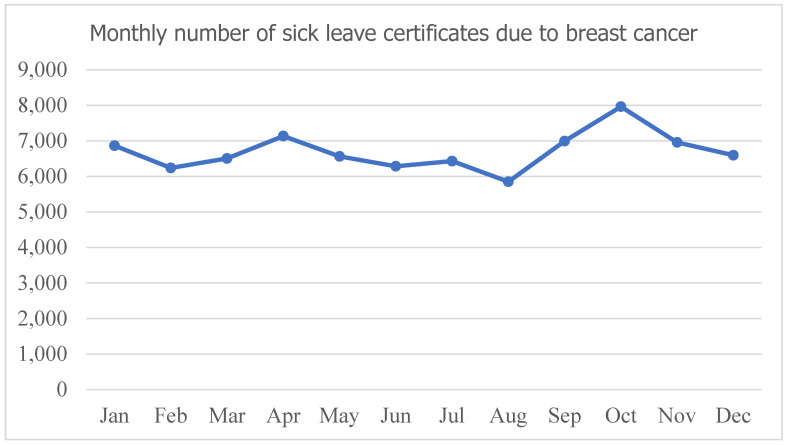
Monthly number of sick leave certificates due to breast cancer (ICD-10: C50), Poland, 2024.

Monthly number of sick leave certificates due to breast cancer in 2024 is presented in Figure 1. The lowest monthly number of sick leave certificates was observed in August and the highest was in October (Figure 1).

Overall rates of sick leave due to breast cancer increased steadily over time among women aged 20–64, with sick leave days per 100,000 females rising from 10,721.5 in 2020 to 12,476.5 in 2024 and the number of certificates per 100,000 females increasing from 456.8 to 693.5 between 2020 and 2024 (Table 2). Rates increased consistently with age, reaching their highest values in women aged 50–54 and 55–59. Younger age groups (20–29) showed very low rates of both sick leave days and certificates across all years (Table 2).

The rates of sick leave days and certificates differed substantially between regions within the same age groups (Table 3). Several regions, including Greater Poland, Silesian and West Pomeranian, had particularly high rates of sick leave days and certificates in the 50–54 age group, with sick leave days exceeding 30,000 per 100,000 and the number of sick leave certificates exceeding 1600 per 100,000 in later years (Table 3).

The cumulative length of sickness absence per year (all medical certificates issued to a particular patient within one year) due to breast cancer (C50) in women was 86.3 days in 2020, 79.6 days in 2021, 74.2 days in 2022, 71 days in 2023 and 68 days in 2024. Overall, the duration of sick leave due to breast cancer decreased over time, from 23.5 days in 2020 to 18.1 days in 2024 (Table 4). This downward trend was observed in all age groups except the two youngest (20–24 and 25–29), which showed greater year-to-year variability without a consistent pattern, supporting the presence of a systemic trend.

The total number of medical certificates for the rehabilitation benefit due to breast cancer varied from 4811 in 2021 to 5281 in 2024 (Table 5). There was a slight increase in the total number of medical certificates granting entitlement to the rehabilitation benefit between 2020 and 2024 (+4.0%) (Table 5).

Social insurance expenditures on sickness absence varied from 112,064.9 thousand PLN (approximately 25.24 million EUR) in 2020 to 207,687.9 thousand PLN (approximately 48.3 million EUR) in 2024 (Table 6). Social insurance expenditures on rehabilitation benefits varied from 56,812.8 thousand PLN (12.8 million EUR) in 2020 to 89,692.6 thousand PLN (20.83 million EUR) in 2024. Overall, there was a substantial increase in social insurance expenditures on sickness absence (+85.3%) and rehabilitation benefits (+57.9%) between 2020 and 2024 (Table 6). The average expenditure per sick leave certificate increased from approximately 2011 PLN in 2020 to 2597 PLN in 2024 (+29.1%), while the expenditure per sickness absence day increased from 85.4 PLN/day in 2020 to 143.7 PLN/day in 2024 (+68.3%). An increase of more than 20% in both expenditures between 2023 and 2024 was observed. The highest absolute expenditures were observed in the Masovian, Silesian, and Greater Poland regions, which are the most populous regions (Table 6).

## 4. Discussion

This descriptive epidemiological nationwide registry-based analysis provides population-level data on sickness absence due to breast cancer among women in Poland. Between 2020 and 2024, a growing burden of sickness absence due to breast cancer was observed. The trend analysis revealed higher frequency of sick leave certificates with relatively shorter durations of absence. In the analyzed years, a slight increase in the number of medical certificates for rehabilitation benefits was observed. Regional differences in the burden of sickness absence due to breast cancer were also observed. Sick leave and rehabilitation benefits related to breast cancer generated significant social insurance expenditures, ranging in total from around 38 million EUR in 2020 to 69.13 million EUR in 2024. This nationwide analysis provides real-life data on sickness absence in breast cancer, which complements clinical data on breast cancer treatment and should be used together to evaluate breast cancer management in Poland [5,6].

Breast cancer generates a substantial economic burden through both direct medical expenditures and indirect costs related to productivity losses and sickness absence [2,15,19]. In 2021, the global value of lost welfare due to breast cancer was estimated at 1.65% of global Gross Domestic Product (GDP) (2538.849 billion USD) [19]. North America was the region with the largest economic losses attributed to breast cancer (557.92 billion USD). In Europe, economic losses attributed to breast cancer in Western Europe were over two times higher than in Eastern Europe (551.35 billion USD vs. 214.788 billion USD) [19]. Łyszczarz B estimated the productivity losses due to work absence attributable to all neoplasms in Poland in 2022 at 969 million EUR [20]. The productivity loss due to breast cancer was estimated at 146.4 million EUR [20]. Seweryn et al. reported that total expenditures for breast cancer (direct and indirect costs) had risen from 305,371,000 EUR in 2017 to 344,649,000 EUR in 2019 [21]. Of these costs, the costs of sickness absence were estimated at 30% of total expenditures [5,21].

To our knowledge, this study is the first nationwide study that presents real-life data on social insurance expenditures borne by the Social Insurance Institution in Poland. This study provided data on public spending on sickness absence related to breast cancer. The Social Insurance Institution (ZUS) is a public institution tasked with social insurance coverage within the social insurance system in Poland. Over 8 million females are covered by social insurance with the Social Insurance Institution (ZUS) in Poland [16]. A significant increase in the social insurance expenditures was observed between 2020 and 2024: from 38 million EUR to 69.13 million EUR. The highest absolute expenditures were observed in the Masovian, Silesian and Greater Poland regions, which are the most populous regions [18]. The reported expenditures may have been influenced by inflation, wage growth, and benefit indexation. Therefore, the observed increase should not be interpreted solely as an increase in the disease burden.

In the event of cancer, the employer covers the cost of sick pay (80% of the base salary) for 33 days for employees under 50, and 14 days for employees over 50. In this study the costs of sickness absence incurred by employers were not included. In Poland, there is a separate public social insurance system for people engaged in agricultural activities—the Agricultural Social Insurance Fund (KRUS). Approximately 600,000–650,000 women in Poland are covered by the KRUS. Because it is a separate social insurance system, sickness absence due to breast cancer in this group was not analyzed.

The rehabilitation benefit is a second short-term benefit, next to sick leave [16]. Obtaining this benefit is possible after using 182 days of sick leave (ZUS ZLA) and requires an opinion from a medical doctor employed by the Social Insurance Institution. Rehabilitation benefits are available to individuals who are expected to return to work within 12 months. The rehabilitation benefit is intended to facilitate a return to work, prevent the need for disability benefits and avoid exclusion from professional activity [16]. Although the number of rehabilitation benefit certificates was an order of magnitude lower than the number of sick leave certificates, total expenditures on rehabilitation benefits were only about two to three times lower than those on sickness absence. This observation suggests that rehabilitation benefits generate substantial social insurance expenditures.

A trend analysis (2020–2024) of sickness absence due to breast cancer, presented in this study showed annual growth (both in absolute numbers and per 100,000 women) in sick leave days and certificates. According to the national cancer registry, between 2020 and 2024 the number of newly detected breast cancer cases increased from 20,6 thousand in 2020, 21 thousand in 2021, to 21,8 thousand in 2022 and 22 thousand in 2023 [7]. It is estimated that the number of newly detected breast cancer cases exceeded 22 thousand cases in 2024. This increase may result from delays in cancer diagnosis during the COVID-19 pandemic (2020–2022) [5,7,8]. The observed patterns in 2020–2021 may additionally reflect reduced healthcare utilization, temporary limitations in access to diagnostic services, delayed treatment initiation, and organizational changes in healthcare delivery during the COVID-19 pandemic. Increased use of remote work during the pandemic period may also have influenced work incapacity patterns among some patients undergoing treatment. An increase in sickness absence may be attributed to the growing number of newly detected cases of breast cancer [7]. The most dynamic growth between 2020 and 2024 occurred in the 45–49 group, where sick leave days increased +33.4% and certificates increased +70.6%. This age range coincides with the starting age for breast cancer screening in Poland (45 years). The eligible population for mammography-based breast cancer screening was expanded to include the 45–49 year old population in November 2023. The observed increase in sickness absence among women aged 45–49 years temporally coincided with the expansion of eligibility for mammography screening in Poland. However, because our dataset does not include information on screening participation, tumor stage, or the mode of detection, this observation should be interpreted only as a temporal association and not as evidence of causality; this observation may reflect multiple overlapping factors, including changes in detection patterns, healthcare access, administrative practices, or healthcare utilization. This observation may reflect multiple factors, including changes in detection patterns, healthcare access, or administrative practices, and should be interpreted with caution [8,10].

Across all age groups, the increase in certificate rates was proportionally larger than the increase in sick leave days, reinforcing the pattern of an increasing frequency of sick leave rather than longer absence duration. An important observation from this study is that despite the increase in sickness absence days and medical certificates, the sickness absence days per certificate decreased from 23.5 days in 2020 to 18.1 days in 2024. Moreover, the cumulative average length of sickness absence per year (all medical certificates issued to a particular patient within one year) due to breast cancer also decreased from 86.3 days in 2020 to 68 days in 2024. The decrease in the sickness absence days per certificate suggests that the increasing burden of sickness absence may reflect a higher number of sickness absence events rather than prolonged absence per episode. The observed decrease in sickness absence duration may reflect multiple overlapping factors, including changes in certification practices, the organization of oncology care, supportive care, treatment pathways, remote work patterns, administrative procedures, and broader healthcare system factors. Updated breast cancer treatment guidelines, access to innovative therapies due to reimbursement, and changes in the functioning of oncology care in Poland may also influence this phenomenon [5,6]. Further analyses are needed to better explain this observation. The observed increase in the number of short-term sick leave certificates may also have implications for healthcare system functioning. The frequent issuance of multiple shorter certificates instead of fewer longer ones may increase the administrative workload for physicians, particularly in oncology care, where visit volumes are already high. This highlights the need for systemic solutions that could reduce the administrative burden while maintaining appropriate support for patients during treatment.

During the analyzed period, Poland experienced substantial inflation and dynamic wage growth. According to Statistics Poland (GUS), annual inflation rates reached 3.4% in 2020, 5.1% in 2021, 14.4% in 2022, 11.4% in 2023, and 3.6% in 2024 [18]. During the same period, the statutory minimum monthly wage increased from 2600 PLN in 2020 to 4300 PLN in 2024 (+65.4%), while the average monthly gross wage in the national economy increased to over 8100 PLN in 2024 [18]. These macroeconomic changes likely contributed substantially to the observed increase in nominal social insurance expenditures related to sickness absence.

There was a slight increase in the total number of medical certificates granting entitlement to the rehabilitation benefit between 2020 and 2024 (+4.0%). This relatively small increase contrasts with the observed increase in sick leave certificates, suggesting that the growth in sickness absence was not accompanied by a proportional increase in long-term work incapacity requiring rehabilitation benefits. Furthermore, no substantial variability in sickness absence due to breast cancer was observed.

The rates of sick leave days and certificates differed substantially between voivodeships within the same age groups, indicating regional variability in the burden of work incapacity related to breast cancer. In this study, the Greater Poland, Silesian, and West Pomeranian regions were identified as the regions with the highest burden of sickness absence due to breast cancer. These differences may reflect multiple overlapping factors, including regional variability in breast cancer incidence, participation in mammography screening programs, healthcare access, the organization of oncology services, employment structure, socioeconomic conditions, physician certification practices, and demographic characteristics of the female population.

The highest burden was observed in highly populated and industrialized regions, which may partly reflect differences in the workforce structure and the number of professionally active women. Regional differences in access to oncology diagnostics, treatment pathways, rehabilitation services, and occupational medicine support may also contribute to variability in sickness absence patterns. However, because the registry-based dataset did not include detailed regional clinical, occupational, or healthcare system indicators, the observed regional differences should be interpreted cautiously. Further studies integrating epidemiological, clinical, occupational, and healthcare system data are needed to better explain these regional patterns.

Importantly, the increasing number of sick leave certificates should not be interpreted directly as an increase in the number of affected individuals or independent clinical episodes of incapacity for work. Because each certificate represented a separate administrative event and continuous episodes could not be reconstructed, the observed patterns may partly reflect continuation certificates, physician certification behaviors, follow-up frequency, or social insurance procedures.

### 4.1. Practical Implications

The findings of this nationwide registry-based study provide clinically relevant information for oncologists, occupational medicine specialists, primary care physicians, and healthcare policymakers involved in breast cancer care. Awareness of age-specific and regional patterns of sickness absence may support clinicians in identifying patient groups potentially requiring prolonged occupational support, rehabilitation, psycho-oncological care, or individualized return-to-work planning during and after cancer treatment. The observed burden of work incapacity additionally highlights the importance of early supportive interventions, symptom control, and multidisciplinary survivorship care aimed at maintaining patients’ functional capacity and their ability to remain professionally active during treatment whenever clinically feasible. The presented data may also support medical certification practice by providing real-world information on the patterns and duration of work incapacity related to breast cancer, potentially facilitating more individualized and evidence-based decisions regarding sick leave and rehabilitation benefit certification.

Data on sickness absence trends may be used to predict the needs of the healthcare and rehabilitation systems. Identifying groups of women with longer sickness absence can help target rehabilitation and psycho-oncological support programs. Data on absence duration and patterns can help employers and public institutions design flexible return-to-work programs that support employees [22]. Integrating data on sickness absence with treatment planning may improve the coordination of surgical, radiotherapy, chemotherapy, and hormone therapy schedules, supporting timely interventions for patients requiring extended leave [11]. The observed increase in sickness absence among women aged 45–49 years highlights the need for further studies examining whether changes in screening policies (extension of the age criterion for screening mammography from 50 to 69 to 45–74 years), earlier diagnosis, treatment patterns, or other factors may be associated with work incapacity outcomes [8,9,10]. The increase in the number of sick leave certificates accompanied by a reduction in the average duration of sickness absence may reflect multiple overlapping factors, including changes in administrative practices, certification patterns, supportive care, treatment organization, healthcare access, remote work patterns, or broader healthcare system changes. This observation requires further analysis. These findings may inform return-to-work planning, occupational rehabilitation, and coordinated care pathways linking oncology services, occupational medicine, primary care, and the social insurance system to support women during and after treatment.

### 4.2. Limitations

This analysis is based on the Social Insurance Institution registry; therefore, the scope of analysis is limited to those data available in the registry. This dataset reflects administrative records of work incapacity and does not include clinical parameters such as disease stage, treatment type, or screening participation. Clinical data (including tumor stage, histopathological type, receptor status and the treatment applied) were not analyzed. Data on sickness absence included both newly detected cases as well as women unable to work due to disease recurrence or adjuvant treatment. Differences in doctors’ behaviors and habits may also influence the differences in the number and length of sick leave. The COVID-19 pandemic (2020–2021), including reduced healthcare utilization, delayed diagnoses, temporary limitations in access to diagnostic and oncological services, changes in healthcare organization, and the increased use of remote work, may have influenced both sickness absence patterns and healthcare-seeking behavior during the analyzed period. Although monthly variability in the number of sick leave certificates was observed in 2024, seasonality was not formally analyzed and these differences should therefore be interpreted cautiously. Finally, our analysis is restricted to insured women registered with the Social Insurance Institution and does not include those outside formal employment, which may slightly affect the generalizability of the findings to the entire population of women affected by breast cancer in Poland. An additional limitation results from the administrative structure of the registry data. Each sick leave certificate was analyzed as a separate administrative event, as continuous episodes of work incapacity could not be reconstructed at the individual level. Therefore, the observed increase in the number of certificates may partly reflect continuation certificates, physician certification practices, or administrative procedures rather than an increase in distinct clinical episodes of sickness absence.

## 5. Conclusions

This population-based registry study revealed a high economic burden of sickness absence due to breast cancer in Poland. Between 2020 and 2024 social insurance expenditures on sickness absence increased by 85.3%, reaching over 200 million PLN (48.3 million EUR) in 2024. The most dynamic growth in sickness absence occurred in women aged 45–49 years. There was an increase in the number of sick leave certificates with a simultaneous decrease in the sickness absence days per certificate. These findings should be interpreted as population-level indicators of the work-disability burden and administrative sickness absence events rather than direct measures of disease incidence or unique patient counts. The findings underline the importance of coordinated return-to-work support, occupational rehabilitation, and the integration of oncology care with social insurance processes.

## Figures and Tables

**Table 1:** Number of sick leave days and sick leave certificates due to breast cancer (ICD-10: C50) among females in Poland, 2020–2024.

	Number of Sick Leave Days Due to Breast Cancer							Number of Sick Leave Certificates Due to Breast Cancer						
	2020	2021	2022	2023	2024	% Change 2024–2020	% Change 2024–2023	2020	2021	2022	2023	2024	% Change 2024–2020	% Change 2024–2023
Overall	1,311,522	1,241,223	1,306,947	1,384,850	1,444,863	10.2	4.3	55,732	56,919	65,240	73,472	79,979	43.5	8.9
≤19	0	31	13	0	0	-	-	0	0	0	0	0	-	-
20–24	221	509	413	365	434	96.4	18.9	15	29	15	18	24	60.0	33.3
25–29	9192	7196	7391	7552	7131	−22.4	−5.6	451	455	413	439	372	−17.5	−15.3
30–34	38,993	38,490	33,952	35,269	38,587	−1.0	9.4	1720	1899	1890	1940	2187	27.2	12.7
35–39	109,557	100,754	105,728	110,967	100,055	−8.7	−9.8	4685	5064	5468	6232	5945	26.9	−4.6
40–44	200,120	184,656	188,117	201,526	210,913	5.4	4.7	8508	8670	9649	11,148	12,150	42.8	9.0
45–49	258,862	249,332	276,269	301,356	345,344	33.4	14.6	11,131	11,858	14,159	16,339	18,993	70.6	16.2
50–54	272,782	261,080	293,232	316,312	325,747	19.4	3.0	11,609	11,824	14,369	16,432	18,098	55.9	10.1
55–59	249,379	243,684	246,862	261,460	255,378	2.4	−2.3	10,477	10,553	11,935	13,454	13,560	29.4	0.8
60–64	121,017	111,552	105,883	101,565	106,049	−12.4	4.4	5097	4782	5182	5194	5910	16.0	13.8
≥65	51,399	43,939	49,087	48,478	55,225	7.4	13.9	2039	1784	2159	2276	2740	34.4	20.4

**Table 2:** Number of sick leave days and sick leave certificates due to breast cancer (ICD-10: C50) per 100,000 females in a given age group, Poland, 2020–2024.

	Number of Sick Leave Days Due to Breast Cancer per 100,000 Females					Number of Sick Leave Certificates Due to Breast Cancer per 100,000 Females				
Year	2020	2021	2022	2023	2024	2020	2021	2022	2023	2024
Overall	10,721.5	10,376.2	11,039.7	11,870.1	12,476.5	456.8	477.8	553.6	632.4	693.5
20–24	23.0	55.1	46.1	41.7	50.3	1.6	3.1	1.7	2.1	2.8
25–29	778.7	643.4	690.2	737.2	726.3	38.2	40.7	38.6	42.9	37.9
30–34	2807.4	2863.7	2596.3	2785.1	3149.4	123.8	141.3	144.5	153.2	178.5
35–39	6871.7	6405.4	6900.1	7514.4	7029.8	293.9	321.9	356.9	422.0	417.7
40–44	13,132.3	12,091.0	12,165.0	12,801.2	13,316.7	558.3	567.7	624.0	708.1	767.1
45–49	19,288.3	17,966.3	19,358.5	20,646.0	23,180.0	829.4	854.5	992.1	1119.4	1274.8
50–54	23,734.0	22,513.6	24,634.6	25,728.1	25,555.3	1010.1	1019.6	1207.1	1336.5	1419.8
55–59	20,926.2	21,172.4	21,830.1	23,430.6	22,975.7	879.2	916.9	1055.4	1205.7	1220.0
60–64	8522.6	8235.5	8190.6	8215.4	8949.6	359.0	353.0	400.9	420.1	498.8

**Table 3:** Number of sick leave days and sick leave certificates due to breast cancer (ICD-10: C50) per 100,000 females by voivodeship and age group, Poland, 2020–2024.

Voivodeship (State/Province) *	Number of Sick Leave Days Due to Breast Cancer per 100,000 Females					Number of Sick Leave Certificates Due to Breast Cancer per 100,000 Females				
Year	2020	2021	2022	2023	2024	2020	2021	2022	2023	2024
Lower Silesian										
40–44	11,123.2	11,177.6	11,658.0	10,149.6	13,025.8	559.5	581.6	625.1	717.5	806.3
45–49	15,782.6	20,747.1	16,686.9	20,423.5	22,295.2	808.8	1057.9	918.9	1153.2	1289.1
50–54	23,001.5	22,953.1	25,637.0	23,590.8	26,583.8	1078.4	1141.1	1446.7	1370.9	1546.8
55–59	21,874.8	22,277.0	18,857.7	21,055.1	22,604.7	942.3	1009.9	951.1	1116.0	1304.4
Kuyavian-Pomeranian										
40–44	15,061.3	15,235.1	16,700.2	15,222.2	13,483.6	504.9	566.3	658.0	704.2	752.6
45–49	18,427.4	19,614.9	22,544.3	20,863.0	23,646.8	670.2	781.5	862.5	858.1	1076.7
50–54	19,536.7	25,105.8	24,617.0	24,114.2	26,108.3	709.8	935.8	1046.0	994.9	1094.1
55–59	18,374.5	18,851.3	23,634.4	22,669.4	23,316.0	631.9	710.8	935.4	908.3	995.5
Lublin										
40–44	10,870.4	10,336.4	11,995.8	11,282.5	12,856.0	431.7	401.2	577.3	646.2	706.2
45–49	18,000.7	17,340.0	12,856.8	14,646.3	20,689.2	681.7	653.2	624.9	746.2	1127.4
50–54	13,956.0	16,494.9	19,363.0	24,474.2	17,535.5	600.8	603.6	789.4	1111.5	912.0
55–59	14,406.8	16,088.3	15,738.7	19,201.8	16,054.3	585.5	640.9	724.4	879.5	744.1
Lubusz										
40–44	11,220.4	12,976.7	13,458.4	15,370.5	14,687.0	549.2	590.0	796.2	867.8	845.2
45–49	21,952.7	19,878.7	16,514.6	24,168.8	22,434.7	886.3	981.6	811.4	1183.7	1197.3
50–54	22,331.7	17,341.3	25,777.3	21,449.4	24,465.1	878.2	802.0	1273.9	1148.2	1339.9
55–59	27,258.1	23,218.5	16,765.9	22,799.2	23,081.0	999.7	948.6	803.3	1122.4	1031.5
Łódź										
40–44	14,835.2	15,311.5	13,128.7	16,306.0	16,261.3	525.8	532.4	538.6	650.1	752.1
45–49	20,339.4	18,973.4	22,558.5	20,544.9	26,465.2	686.6	740.7	956.0	961.1	1213.1
50–54	24,577.2	22,004.9	26,799.0	25,924.3	28,987.0	836.9	807.6	1038.1	1078.8	1250.0
55–59	23,156.4	18,648.7	24,086.2	23,719.5	29,481.6	780.8	710.3	935.5	998.6	1255.3
Lesser Poland										
40–44	11,659.1	10,590.0	9531.6	12,307.7	12,193.9	594.4	567.7	557.3	706.5	706.3
45–49	16,987.5	15,413.1	15,381.2	18,469.5	18,524.3	868.0	830.5	852.6	1116.1	1202.4
50–54	22,343.9	20,884.7	20,919.7	20,856.4	23,898.0	1163.2	1074.2	1177.0	1238.9	1387.4
55–59	17,966.0	18,629.5	20,217.9	24,440.8	22,449.8	902.1	967.3	1129.2	1334.7	1330.5
Masovian										
40–44	14,230.7	11,862.8	11,844.5	11,589.8	12,642.3	582.3	605.8	616.8	671.3	766.1
45–49	22,165.5	18,009.1	21,659.6	22,133.0	23,522.6	919.9	886.5	1193.5	1286.8	1388.8
50–54	24,812.5	21,235.0	23,719.7	24,313.7	26,670.2	981.9	955.0	1199.7	1295.0	1581.6
55–59	24,800.0	23,261.7	23,873.7	23,550.8	23,598.6	988.3	920.3	1163.6	1351.1	1355.0
Opole										
40–44	8418.6	6630.2	10,380.0	10,131.0	11,091.9	440.9	377.1	506.5	524.9	757.1
45–49	14,491.4	11,957.0	16,141.2	18,513.2	17,718.3	758.5	642.5	834.3	967.6	1020.8
50–54	19,638.5	18,166.9	20,472.5	21,008.2	16,810.4	1001.5	963.1	1025.8	1141.6	1009.7
55–59	18,320.8	14,440.4	21,734.9	27,074.3	23,228.0	770.4	751.0	1124.2	1424.1	1429.3
Subcarpathian										
40–44	9985.1	9941.3	8089.7	11,539.6	9464.6	437.5	407.8	416.2	655.3	507.4
45–49	14,792.6	12,870.8	18,109.1	19,711.7	19,861.2	623.7	606.2	928.8	972.3	1066.8
50–54	21,957.5	18,163.8	19,237.3	20,381.0	20,096.7	853.3	764.6	839.1	937.0	1058.2
55–59	16,557.7	19,373.6	17,185.4	19,002.1	16,314.0	700.2	816.3	829.1	921.1	845.2
Podlaskie										
40–44	11,354.7	10,629.3	9897.6	13,114.9	14,522.7	336.9	306.7	404.0	582.2	698.6
45–49	24,034.9	17,619.0	18,174.4	18,190.9	26,275.5	746.1	571.6	730.1	834.9	1086.3
50–54	21,861.2	18,326.4	28,078.1	21,116.4	23,364.5	800.9	706.3	1106.4	1024.0	1155.0
55–59	24,444.3	16,525.9	17,595.5	20,951.8	19,376.6	823.5	656.3	703.9	1090.5	1160.4
Pomeranian										
40–44	12,611.5	11,519.9	11,781.2	12,105.3	13,892.5	526.2	592.9	685.3	820.2	870.9
45–49	19,167.1	19,620.4	18,118.1	20,182.8	21,358.4	813.5	874.9	943.0	1112.9	1212.1
50–54	27,740.8	24,621.3	23,114.4	28,074.8	26,481.4	1129.5	1084.7	1222.7	1444.4	1371.0
55–59	21,512.7	23,899.9	27,004.5	23,904.1	22,839.1	853.1	1033.5	1257.8	1236.5	1202.9
Silesian										
40–44	14,478.5	10,876.0	12,015.2	12,625.0	11,177.1	649.8	604.3	678.3	747.4	757.0
45–49	18,577.9	16,932.4	20,188.4	21,550.3	24,379.8	934.8	947.5	1124.9	1322.4	1531.1
50–54	24,689.0	22,883.3	23,583.1	30,758.3	27,457.6	1195.9	1300.8	1321.0	1788.6	1793.9
55–59	20,079.0	20,755.0	19,028.2	20,636.7	21,473.4	995.1	1056.0	1081.1	1192.0	1320.1
Świętokrzyskie										
40–44	13,052.7	10,686.8	9297.6	11,597.8	10,372.0	368.3	415.1	359.0	490.5	517.6
45–49	14,904.8	13,746.7	15,912.4	18,494.2	22,068.9	545.3	552.5	616.4	804.9	974.2
50–54	21,284.8	19,874.8	23,840.8	18,142.7	20,572.3	818.9	666.4	977.4	829.9	1030.1
55–59	21,243.8	19,280.0	20,441.3	25,578.4	23,359.7	855.3	713.6	884.3	1047.6	1046.6
Warmian-Masurian										
40–44	10,816.9	13,434.2	12,941.7	9998.2	10,031.5	430.9	503.5	542.6	488.2	519.7
45–49	19,657.1	13,560.7	15,387.2	16,589.1	17,900.9	729.4	561.9	576.8	692.8	860.8
50–54	20,933.3	23,255.5	20,105.5	25,519.4	16,683.1	853.9	945.9	855.7	1011.9	818.6
55–59	14,674.8	21,995.4	19,647.5	21,249.0	22,821.4	603.8	826.2	884.7	899.2	1040.3
Greater Poland										
40–44	15,438.8	15,191.2	16,504.8	14,035.1	19,140.0	698.5	747.8	879.9	781.2	1037.9
45–49	21,098.1	21,311.4	23,633.4	23,546.1	26,645.0	954.8	1072.8	1342.5	1355.1	1449.8
50–54	29,836.0	28,682.9	32,484.3	31,662.5	33,705.0	1286.0	1267.7	1665.9	1753.5	1878.7
55–59	21,155.3	25,677.9	27,853.7	26,804.5	26,801.7	983.5	1089.9	1376.5	1469.2	1411.8
West Pomeranian										
40–44	13,355.9	11,857.4	5207.3	16,615.5	12,689.2	575.4	638.1	337.8	881.2	751.6
45–49	20,757.4	17,471.9	10,351.0	19,866.5	26,511.4	910.7	846.5	581.4	1084.5	1338.1
50–54	23,048.9	25,686.5	15,905.8	31,751.0	22,935.0	968.2	1188.6	752.3	1684.9	1486.3
55–59	22,989.1	21,270.7	14,459.5	29,133.5	22,896.0	939.2	899.0	695.8	1572.1	1133.7

* Voivodeship corresponds to an administrative region in Poland; for clarity, it may be referred to as State/Province for international readers.

**Table 4:** Sickness absence days per certificate due to breast cancer, 2020–2024, Poland.

Sickness Absence Days per Certificate Due to Breast Cancer					
Year	2020	2021	2022	2023	2024
Overall	23.5	21.8	20.0	18.8	18.1
≤19	-	-	-	-	-
20–24	14.7	17.6	27.5	20.3	18.1
25–29	20.4	15.8	17.9	17.2	19.2
30–34	22.7	20.3	18.0	18.2	17.6
35–39	23.4	19.9	19.3	17.8	16.8
40–44	23.5	21.3	19.5	18.1	17.4
45–49	23.3	21.0	19.5	18.4	18.2
50–54	23.5	22.1	20.4	19.2	18.0
55–59	23.8	23.1	20.7	19.4	18.8
60–64	23.7	23.3	20.4	19.6	17.9
≥65	25.2	24.6	22.7	21.3	20.2

**Table 5:** Number of medical certificates granting entitlement to rehabilitation benefit due to breast cancer (ICD-10: C50), Poland, 2020–2024.

Number of Medical Certificates Granting Entitlement to Rehabilitation Benefit Due to Breast Cancer							
Voivodeship (State/Province)	2020	2021	2022	2023	2024	% Change 2024–2020	% Change 2024–2023
Overall	5076	4811	4896	5208	5281	4.0	1.4
Lower Silesian	419	409	404	366	398	−5.0	8.7
Kuyavian-Pomeranian	279	272	304	322	333	19.4	3.4
Lublin	176	192	189	213	233	32.4	9.4
Lubusz	136	120	124	123	134	−1.5	8.9
Lodz	347	303	317	286	348	0.3	21.7
Lesser Poland	401	382	396	438	431	7.5	−1.6
Masovian	787	680	700	707	700	−11.1	−1.0
Opole	138	113	150	185	142	2.9	−23.2
Subcarpathian	181	195	177	214	178	−1.7	−16.8
Podlaskie	149	92	112	122	153	2.7	25.4
Pomeranian	393	387	364	407	399	1.5	−2.0
Silesian	743	715	676	754	765	3.0	1.5
Swietokrzyskie	113	98	119	122	122	8.0	0.0
Warmian-Masurian	166	182	175	219	224	34.9	2.3
Greater Poland	493	477	510	513	510	3.4	−0.6
West Pomeranian	155	194	179	217	211	36.1	−2.8

**Table 6:** Social insurance expenditures (in PLN thousand) on sickness absence and rehabilitation benefits in 2020–2024, Poland.

Social Insurance Expenditures (in PLN Thousand) on Sickness Absence							
Voivodeship (State/Province)	2020	2021	2022	2023	2024	% Change 2024–2020	% Change 2024–2023
Overall	112,064.9	121,670.7	145,758.7	172,582.7	207,687.9	85.3	20.3
Lower Silesian	7999.0	9979.1	10,773.1	12,098.6	15,721.8	96.5	29.9
Kuyavian-Pomeranian	5907.4	6913.5	8776.6	9283.5	11,222.8	90.0	20.9
Lublin	4733.7	5356.1	6202.5	7716.7	8552.7	80.7	10.8
Lubusz	2872.8	3170.7	3453.8	4584.3	5446.4	89.6	18.8
Lodz	8230.2	8471.9	10,465.8	11,689.3	15,455.7	87.8	32.2
Lesser Poland	8988.3	9712.4	11,134.0	14,361.4	17,186.3	91.2	19.7
Masovian	18,396.5	18,617.7	23,046.3	26,193.6	31,960.0	73.7	22.0
Opole	2460.2	2301.3	3437.7	4228.4	4365.0	77.4	3.2
Subcarpathian	4939.1	5324.1	6330.7	7988.3	8948.5	81.2	12.0
Podlaskie	3508.2	3309.7	4159.2	4653.1	6197.6	76.7	33.2
Pomeranian	7136.1	7702.8	8884.6	10,836.7	13,118.7	83.8	21.1
Silesian	13,425.0	14,125.2	16,714.2	21,047.6	24,280.8	80.9	15.4
Swietokrzyskie	3216.2	3289.9	4097.9	4642.4	5500.3	71.0	18.5
Warmian-Masurian	3445.5	4260.4	4622.5	5210.6	5864.1	70.2	12.5
Greater Poland	11,553.1	13,620.7	17,050.3	19,054.7	24,389.0	111.1	28.0
West Pomeranian	5253.7	5515.3	6609.6	8993.4	9478.3	80.4	5.4
**Social insurance expenditures (in PLN thousand) on rehabilitation benefits**							
**Overall**	**56,812.8**	**57,275.9**	**63,658.3**	**74,320.0**	**89,692.6**	**57.9**	**20.7**
Lower Silesian	4629.7	4803.3	5218.3	5208.4	6392.8	38.1	22.7
Kuyavian-Pomeranian	3108.5	3233.4	3044.5	4864.9	5380.9	73.1	10.6
Lublin	1951.1	2249.3	2027.8	2954.7	3790.7	94.3	28.3
Lubusz	1708.6	1405.8	1347.6	1978.9	2634.2	54.2	33.1
Lodz	4166.7	3690.3	3273.0	4383.9	6601.7	58.4	50.6
Lesser Poland	4442.3	4498.7	4485.5	6390.3	7228.1	62.7	13.1
Masovian	8697.2	8001.5	14,950.1	10,018.4	11,436.5	31.5	14.2
Opole	1521.2	1323.8	1522.0	2556.1	2280.9	49.9	−10.8
Subcarpathian	1995.2	2284.5	2196.0	2954.7	2907.3	45.7	−1.6
Podlaskie	1642.4	1077.8	1354.9	1676.6	2505.7	52.6	49.5
Pomeranian	4354.1	4568.9	3601.2	5593.2	6473.2	48.7	15.7
Silesian	8234.2	8411.5	7304.0	10,389.4	12,657.2	53.7	21.8
Swietokrzyskie	1344.8	1487.8	1043.7	1800.3	2361.2	75.6	31.2
Warmian-Masurian	1829.8	2155.6	1823.7	3064.6	3598.0	96.6	17.4
Greater Poland	5445.4	5635.0	8603.6	7311.1	8529.2	56.6	16.7
West Pomeranian	1741.6	2448.5	1862.3	3174.5	4915.1	182.2	54.8

## Data Availability

Data are available from the corresponding Author upon reasonable request.
